# Societal context-dependent multi-modal transportation network augmentation in Johannesburg, South Africa

**DOI:** 10.1371/journal.pone.0249014

**Published:** 2021-04-08

**Authors:** Thembani Moyo, Alain Y. Kibangou, Walter Musakwa

**Affiliations:** 1 Department of Urban and Regional Planning, Future Earth and Ecosystems Services Research Group, Doornfontein Campus, University of Johannesburg, Johannesburg, South Africa; 2 Gipsa-Lab, CNRS, Inria, Univ. Grenoble Alpes, Grenoble, France; Univ. Lyon, ENTPE, Univ. Gustave Eiffel, FRANCE

## Abstract

In most developing countries, formal and informal transportation schemes coexist without effective and smart integration. In this paper, the authors show how to leverage opportunities offered by formal and informal transportation schemes to build an integrated multi-modal network. Precisely, the authors consider integration of rickshaws to a bus-train network, by taking into account accessibility and societal constraints. By modelling the respective networks with weighted graphs, a graph augmentation problem is solved with respect to a composite cost taking into account constraints on the use of rickshaws. The solution, is based on finding a minimum cost spanning tree of a merged graph. The method is applied in the South African context, in the city of Johannesburg where rickshaws are not yet a significant part of the transportation system. The implications of the study reveal that using non-motorised transportation services is a viable option of improving mobility in the city. The composite cost introduced herein could be used for new routing algorithm including societal, environmental, architectural contexts and commuter experiences through rating.

## Introduction

Mobility in urban spaces is continuously transforming and adapting to daily challenges, which has led to promotion of green mobility modes such as bicycle sharing services [1] and non-motorized or electrical rickshaws [2]. From an environmental perspective these forms of mobility lead to a reduction in traffic congestion whilst also promoting healthy living [3, 4]. In the developing world, transportation planners are faced with the challenge of managing the mobility systems with limited resources [5, 6]. This has led to emerging modes such as tuk-tuks and rickshaws filling a void in the current public transportation system. By linking commuters to their points of interest and acting as feeders to the larger public transportation networks, they play an important role in the first and last mile of commuting [7, 8].

These modes have over the years proliferated as a sustainable mobility alternative and a viable solution to potentially reduce the carbon footprint due to public transportation activities [7, 9]. Notable examples include the pedicab in the Philippines, becak in Indonesia, cycle-rickshaw in Bangladesh and tuk-tuk in Thailand. In Delhi, India, and Jakarta, Indonesia, these modes were initially restrained to subdue their proliferation because they were considered chaotic. However there is evidence that restraining these modes has not improved mobility flow [10]. On the contrary, there is evidence that rickshaws are eco-friendly and a cheap alternative for low-income commuters. In Bogota, Colombia, commuters during the peak hours prefer using pedicabs from a time-saving perspective, although the trip fare would be more expensive when compared to feeder bus [11, 12].

Although not globally recognized as an official public transportation mode, if non-motorised transportation (NMT) such as rickshaws and tuk-tuk are planned for properly and modernized they can play a vital role in the city’s mobility network by acting as feeders to the larger public transportation network [8, 9, 13, 14]. Besides having a positive ecological footprint and improving connectivity in urban areas, the integration of such green mobility services would serve to create new employment and income opportunities [15]. However, in developing countries, these mobility services are operated in an informal way. Indeed, tuk-tuk and rickshaw operators gather at specific strategic locations wherever commuters are likely to be found, usually near commercial businesses districts [2]; a similar pattern with motorcycle taxis operators (which can be found in several African countries) has been also observed in [16]. To control the movement patterns of tuk-tuks, it is crucial to rethink the design of mobility systems, as these informal green mobility modes have the capacity to cater for short-trips, create employment opportunities, whilst only requiring low-start up capital and low-maintenance [15, 16].

Physical integration [17, 18] of a bus network, train network and non-motorised network is a challenging task [19, 20]. Operational constraints of both networks are to be taken into account to make the integration successful. However, questions related to accessibility, cost, security, profile of commuters are to be included in the design of the integrated system. In this paper, these constraints are referred to as *societal constraints*. Consequently the aim of the study is to introduce a methodological approach to merge an existing motorized formal network with a non-motorized network to be designed. The merging is obtained by minimizing a societal cost function which includes various parameters. The proposed method is illustrated in the South African context, in the City of Johannesburg, where rickshaws and tuk-tuks can still be considered as new forms of public transportation. Indeed, the first tuk-tuk started to operate in 2010 in Sandton and in 2013 within Melville surburb in very specific routes. Johannesburg has only few dozens of registered tuk-tuks drivers operating under auspices of a private company [15] and only 12% of commuters use rickshaws to connect with other modes of transport. Adoption of this NMT mode is impaired by accessibility and safety issues.

## Related works

### Integration of non-motorized transportation modes with motorized modes

Recent studies on use of multi-modal mobility have focused on the point of interchange [4, 8, 17]; road network spatial design [21, 22]; or transport and social development [23]. Generally commuters utilize numerous modes when the main public transportation system stop or station is not available within walking distance or when NMT alone is not a viable option (such as for long trips). Therefore, transfer between two or more modes becomes inevitable. These interchanges have become common practice in many large cities. However, currently most public transportation systems in developing countries are not connected, as they are run as separate entities and do not share synergies. Literature on interconnection of mobility systems has shown there are numerous merits to this practice however its implementation involves complex details [3, 24].

Wang et al. [25] assessed commuting data along the China multi-modal corridor in Yong-Tai-Wen, with key emphasis on travel preference trends. The study reveals that long distance trips traditionally trigger the use of more than one mode of transportation. Also commuters are less elastic in their travel time and trip cost, with most commuters preferring modes that reduce travel time on business trips.

Wang et al. [14] developed an algorithm to provide automated matching for a high occupancy vehicle ridesharing system. The study sought to minimize the total passenger ride time, total travel distance of the vehicle, the total toll fee, and the total taxi service cost whilst also ensuring requests that cannot be fulfilled by the ridesharing vehicle were assigned an alternative mode of transportation. Wang et al. [14] also outlined in their algorithm how the matching of the drivers and riders in ridesharing system was viewed as a pickup and delivery problem. The results reveal, as a participant in ridesharing becomes more flexible in time, the less one should pay for his/her trip.

Rahman et al. [8] unpacks the popularity of rickshaws as a response to an existing transportation gap, as they are demand responsive and more suitable for short commuting trips. It is worth noting that there are currently few existing public transportation systems which are linked with rickshaws. Few contemporary research works on integrating rickshaws with the larger public transport network can be found in the literature [8, 10, 23].

Sagaris and Arora [23] evaluated how public transportation and cycling services can become complementary, with NMT providing feeder services to public transportation. Using distance to identify the ecological niche of both modes, they assessed the modes based on sustainability indices namely safety, health, happiness and equity. Results from their work reveal how the integration of the two modes has the potential to reinforce the city authority’s goal for more efficient energy use and reduced pollution, whilst also addressing the inherent challenge of traversing the first and last mile of the trip.

Sobhani et al. [10] assessed three modes namely the rickshaws, bicycle sharing services and Human haulers for competitiveness using a multi-criteria decision making analysis. Findings from the evaluation highlight the need to re-brand these modes from informal to formal, as this regularization will have a symbiotic benefit to city authorities and the operators. Also the allocation of designated routes will prevent disputes between the operators.

Whilst Rahman et al. [8] proposed how the physical planning design of the interchange area between the two modes can be enhanced by placing rickshaw stands within close proximity of bus stops. Results from their work reveal challenges of creating an integrated payment system, as traditionally rickshaws rely on a bargaining process based on the route and destination. Harding et al. [26] have outline how this bargaining process has led to criticism and negative perceptions of rickshaws. However through formalising operations of rickshaws there is a possibility to have a fixed rate based on the distance [27].

In contrast, to these works, this paper considers the case where a motorized transportation operator aims to extend its network by including non-motorised rickshaws. The problem of interest is therefore, which new routes to create and which number of NMT to be included in the network according to the actual demand for transportation. This problem is similar to the one tackled by Jin et al. [9]. In a bid to mitigate transportation problems with a limited budget Jin et al. [9] proposed the improvement of the public transportation systems in order to attract more ridership, through the introduction of green technology based modes such as bicycle-sharing. The problem of determining bicycle-sharing station location and path network design was defined as being a graph, where the set of nodes represented the stations and the links represented the cycling lanes between the nodes and the factors influence travel demand as the set of constraints. Using a two-stage nonlinear stochastic programming model the nodes and links were determined for the feasible demand scenarios. Although the model was able to determine optimal locations of bicycle-sharing stations and cycle lanes, the modal is limited in that it does not consider the road network in determining the cycle path nor constraints that could prevent adoption of cycling on some specific areas (safety, pollution, steepness). In this paper, these constraints are specifically integrated through a composite cost function. It is worth noting that by solving the transportation problem in the proposed framework, uncontrolled proliferation of NMTs is prevented and for commuters a centralized payment system is provided. This is particularly challenging for developing countries. Having a centralized payment system makes it easier for the commuter to pay for trips online or through a mobile application. In Seville, Spain an integrated fare system allows commuters to utilize bike sharing services on the same ticket as the train or bus [23]. Besides providing security, online payments also act as a platform were promotional coupons can be in-cooperated in the payment system which can be used to encourage more ridership during off peak hours.

The framework proposed in this paper includes the following steps:

1. Given the existing motorized network, societal and road constraints, determine stations and routes for rickshaws to improve connectivity and travel time.
2. Determine number of rickshaws operators per routes according to the demand.
3. Devise an integrated and secure payment.

In this paper, the focus is on the first step which can be viewed as a network augmentation problem.

### Network augmentation problem

Improving accessibility in transportation planning can be viewed as a problem of economically improving a network to meet given survivability requirements, a problem which occurs in a number of areas. For instance, a problem of this type is concerned with creating more connections in a computer network so that it survives the failure of a given number of cables or terminals [28]. Similar problems arise in statistics [29] and data security [30]. It is natural to model these networks by graphs or directed graphs and use graph connectivity parameters to handle the survivability requirements.

The problem to be solved is then a graph augmentation problem [31], which seeks to suggest new edges / short-cuts that, once added to an input graph, will improve a specific property of the graph such as the overall connectivity of the nodes or a given total cost. In connectivity augmentation, one wishes to add a minimum cost set of new edges to a graph to satisfy certain connectivity requirements, for example, k-edge-connectivity. A graph is said to be *k*-edge connected if it remains connected whenever fewer than *k* edges are removed. This *k*-edge connectivity augmentation problem can be solved by a minimum cost spanning tree algorithm for *k* = 1. It becomes NP-complete for every fixed value *k* ≥ 2. There is a vast and expanding literature on approximation algorithms for various connectivity requirements; see [32] for a survey. In contrast to this literature, our graph augmentation problem is related to a composite cost that includes a societal cost, geographic location cost, and mobility cost in a developing country setting. It is shown in the sequel that the minimum composite cost is obtained through finding a minimum spanning tree. Therefore, improvement of the connectivity comes as a by-product.

#### Analysis of transportation networks using centrality measures

Ensuring high connectivity and integration in networks is essential to prevent network failure. Generally in large networks, such as public transportation networks, the nodes or bus stops with the most spreading ability are called influential nodes [33]. Consequently the identification of such influential nodes and ranking them based on their spreading ability is of vital importance, as this will ensure high connectivity in the network. Scholars have explored the problem of identifying the influential nodes in networks by determining either the degree centrality, betweenness centrality or closeness centrality of nodes [33, 34]. These metrics have been used in analyzing social networks, communication networks, power networks and transportation networks [35, 36]. The centrality score highlights nodes whose failure will have the greatest consequences on the whole system. Networks with many influential nodes survive better failures along the network than networks with few influential nodes.

Whilst the degree centrality measures how connected a vertex is by counting the number of direct links each vertex has to others in the network. It is usually represented by a degree distribution. Nodes with higher degree are more central. One notable drawback of the degree centrality is that it does not capture an important aspect of centrality as it does not take into consideration the structure of the whole network [33]. As one node that has fewer high influential neighbours could have a higher influence in the network in relation to a node with greater number of less influential neighbours.

In response to this drawback the use of closeness centrality was proposed in literature as it measures the number of steps on average, from any node to reach every other node in the network of a connected graph [37]. Also it is noteworthy to highlight how closeness centrality is an index defined in terms of distance as it measures the steps taken in-between the nodes. Defining by *d*(*i*, *j*) the distance between vertices *i* and *j*, the closeness of a vertex *i* is computed as $C_{c}\left(i\right)=\frac{n-1}{\sum_{j\in ⊑}d\left(i,j\right)}$, *n* being the number of vertices in the graph. In this work, the distance will be computed according to the travel time between nodes. It should be noted that though highly efficient in determining the influential nodes, closeness centrality due to the computational complexity involved in calculations, is not easily applicable in large-scale networks [38].

Lastly betweenness centrality is commonly used to help distinguish the level of influence a node has over the flow of information in a network or how often a node serves as a bridge from one part of the graph to another [39]. By measuring the betweenness centrality the influential nodes are identified.

## Materials and methods

**Fig 1:**
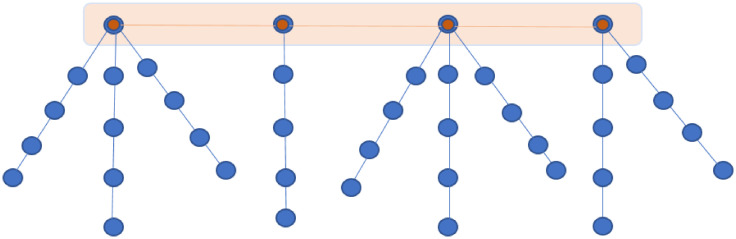
Graph *G* representing a bi-modal integrated network with the path graph (train network) in orange and the forest (bus network) in blue.

The paper is concerned with an integrated public transportation network including two modes: train and buses. Inspired by topologies of train-buses networks that can be encountered in developing countries (see the case study in the sequel) the following model is adopted. The train network is modeled with a weighted path graph $P(V_{P},E_{P},W_{P})$; $V_{P}$, *E*_*P*_, and *W*_*P*_ standing for the vertex (nodes or stations) set, the edges set, and the weights set, respectively. The bus network is represented as a weighted forest $F(V_{F},E_{F},W_{F})$, where $V_{F}$, *E*_*F*_, and *W*_*F*_ represent the vertex (nodes or stations) set, the edges set, and the weights set, respectively. Precisely, the bus network is a union of disjoint rooted trees where the roots of each tree allow connection to the graph *P*. The integrated bi-modal network is therefore represented by G(V,E,W)=P∪F, a connected graph which can be viewed as a tree (see Fig 1 for an illustration).

**Fig 2:**
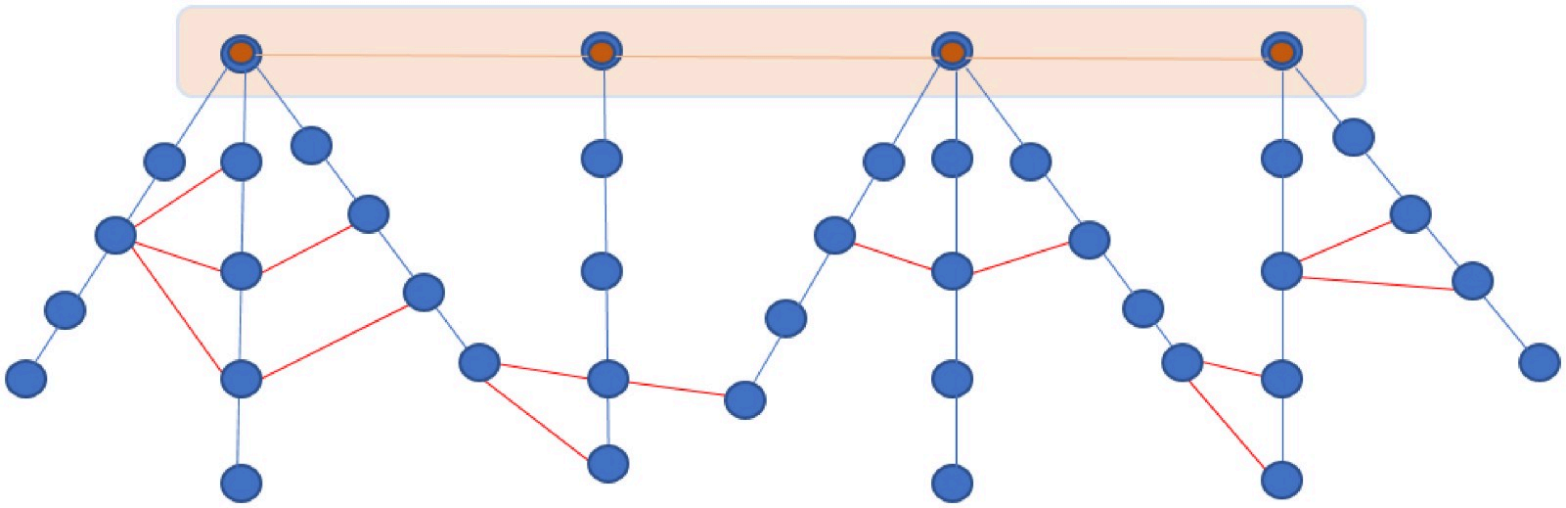
Graph representing a multi-modal integrated network with train (orange links), bus (blue links) and non-motorized modes (red links).

Due to the structure, any link failure in *G* will create isolated components. The problem is to augment the graph *G* by adding new edges to make the graph more robust and to reduce the distance between nodes in disjoint trees of *F*. The new edges will represent a third transportation mode (See Fig 2), a non-motorised rickshaw network precisely.

The graph $G_{NMT}\left(V,E_{NMT},W_{NMT}\right)$ represents the rickshaw network. Our problem is: from $G_{NMT}\left(V,E_{NMT},W_{NMT}\right)$ and G(V,E,W), find an augmented graph with minimum cost. Usually the cost is related to travel time or connectivity metrics. Herein a composite cost is adopted. It includes operational, accessibility and societal constraints. This cost is the sum of weights defined hereafter.

**Definition**: Consider a multi-modal transportation network represented with a weighted graph. The edge weight between vertices *i* and *j* using mode *m* is given by:

$$\begin{array}{c} w_{ijm}=t_{ijm}a_{ijm}s_{ijm}p_{ijm}, \end{array}$$ (1)

where

- *t*_*ijm*_: represents the travel time between *i* and *j* when using mode *m*.
- *a*_*ijm*_: represents the accessibility cost between *i* and *j* using mode *m*. For a motorized mode *m*, if there is a direct link between *i* and *j*, then *a*_*ijm*_ = 1, else *a*_*ijm*_ = ∞. For a NMT mode if the distance between *i* and *j* is lower than a given threshold *D* and there is a cycling path then *a*_*ijm*_ ≥ 1 else *a*_*ijm*_ = ∞. This cost can also take into account steepness of the link between *i* and *j* in order to consider the physical effort required to rickshaw operators.
- *s*_*ijm*_: represents the security (safety) cost between *i* and *j* using mode *m*. It allows for the taking into account the crime level in the traversed zone. We assume the motorized mode as being safe. Therefore, *s*_*ijm*_ = 1 for motorized modes. For NMT, *s*_*ijm*_ ≥ 1. Here, various perception of safety can be included. One can for instance set the cost to ∞ if the link is not safe at all from a statistical point of view or from commuters perception.
- *p*_*ijm*_: represents a preference parameter which is equal to 1 for motorized modes since it is the existing one. For NMT, *p*_*ijm*_ = ∞ if there already exists a motorized link between *i* and *j* else *p*_*ijm*_ ≥ 1. This parameter allows not adding NMT on a route already served by a bus and also to take into account the potential demand on NMT.

**Proposition 1**: Given the societal cost (1), the augmented network with minimal cost is given by $G\cup T_{G\cup G_{NMT}}$, where $T_{G\cup G_{NMT}}$ stands for the minimum spanning tree of *G*∪*G*_*NMT*_.

*Proof*: *By the definition of the weights in* (1) *and the problem definition*, *G* *is necessarily a sub-graph of the augmented one*. *Consider G* ∪ *G*_*NMT*_ *the union graph of motorized and NMT modes*. *It is known that* $T_{G\cup G_{NMT}}$ *is the minimum-cost sub-graph connecting all vertices, since the weights are positive*. *By adding to this minimum spanning tree the remaining edges of* *G*, *the augmented graph is obtained with G as sub-graph having minimum cost*.

It is worth noting that the solution does not depend on the structure of the original graph *G*. Therefore, the solution can be applied to more general networks. Several algorithms for computing minimum spanning trees are available in the literature, including Kruskal, Prim, and Boruvska algorithms [40–42]. Recent algorithms exhibit a linear time execution (linear in the number of edges). Parallel scalable algorithms have been also designed to tackle large and dense graphs (see for instance [43]. the following proposition, the authors define a lower bound of number of NMT links that can be added to the network.

**Proposition 2**: Let *e*_*G*_ be the edge of minimal weight in *G*. Define $A_{NMT}$ the set of edges in *G*_*NMT*_ with a weight lower than the weight of *e*_*G*_ and $B_{NMT}$ the set of NMT edges added in the augmented graph then

$$\begin{array}{c} |B_{NMT}|\geq |A_{NMT}|, \end{array}$$ (2)

|X| standing for the cardinality of the set |X|.

*Proof*: *For constructing a minimum spanning tree using Kruskal’s algorithm for instance, one start adding the edges of* *G* ∪ *G*_*NMT*_ *in increasing order*. *Therefore, the first edges to be added will be the ones in* $\left|A_{NMT}\right|$ *except those inducing cycles*. *Therefore* $\left|A_{NMT}\right|$ *is a lower bound of NMT edges in the augmented graph*. order to increase the connectivity of the original graph, it is then necessary to make $\left|A_{NMT}\right|$ as big as possible. Since the weights result in the product of different parameters, one can act independently on the travel time, accessibility, security, and preference to minimize the composite weight of an NMT edge. Precisely, for each NMT edge, one would like to set $t_{ijm}a_{ijm}s_{ijm}p_{ijm}\leq \bar{w}$, $\bar{w}$ being the weight of *e*_*G*_. There are four degrees of freedom to meet the weight of the NMT links as lower than $\bar{w}$. There is potentially an infinity of solutions by working on each parameter. However, for some networks, urban planners cannot really work on all the parameters. Assume that the only degrees of freedom are accessibility and security. Then efforts must be made to get $a_{ijm}s_{ijm}\leq \frac{\bar{w}}{t_{ijm}p_{ijm}}.$ Working jointly on security and accessibility in order to meet the above constraint could require less investment than working on a single item.

Taking into account the weights definition, a set of admissible edges in *E*_*NMT*_ is defined. Therefore, to solve the problem, the approach is divided into 3 steps.

**Proposed method**:

- Step 1: Given collected data, perform a social analysis, a geographical analysis, and a mobility analysis to get parameters *s*_*ijm*_, *a*_*ijm*_, and *p*_*ijm*_ for the NMT mode. Build the set *E*_*NMT*_ of candidate edges (short-cuts) by discarding edges with infinite weights; then compute the finite weights in *W* and *W*_*NMT*_;
- Step 2: Find a minimum weight spanning tree on the graph *G*∪*G*_*NMT*_, with corresponding weights *W*∪*W*_*NMT*_.
- Step 3: Augment the original graph with edges in the spanning tree.

**Fig 3:**
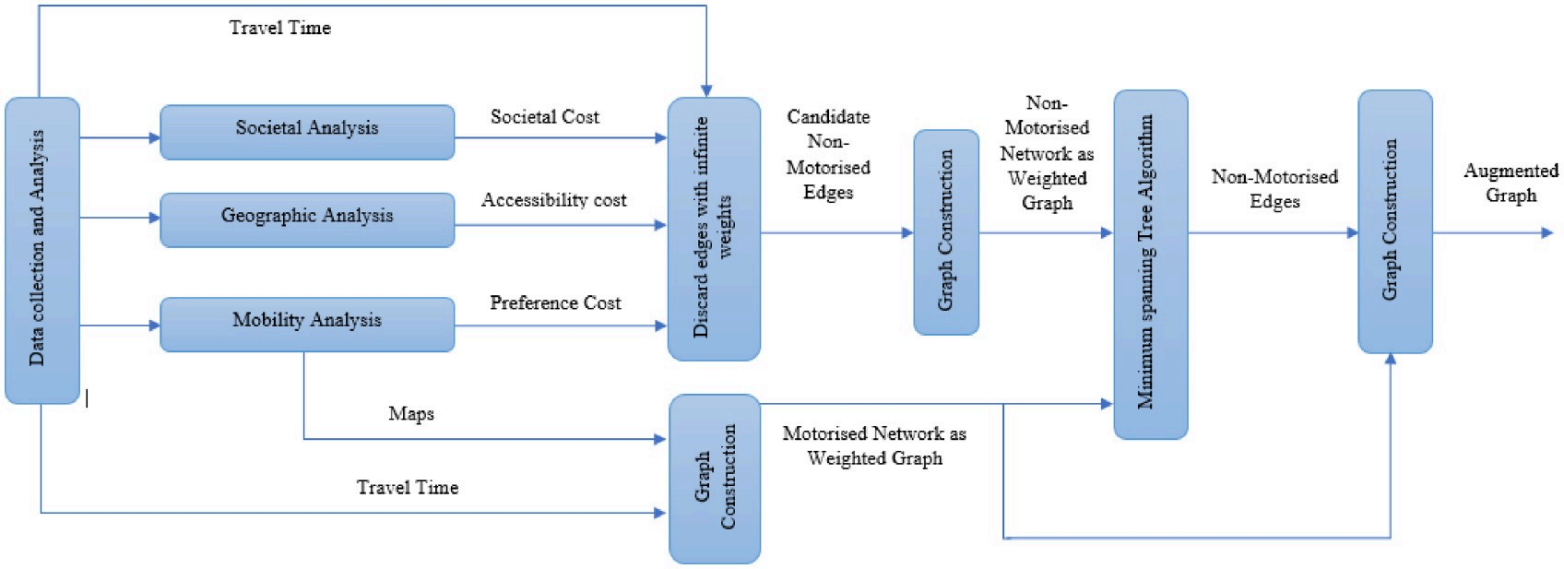
Flowchart of the proposed method.

The proposed method is summarized by the following flowchart in Fig 3.

### Example

**Fig 4:**
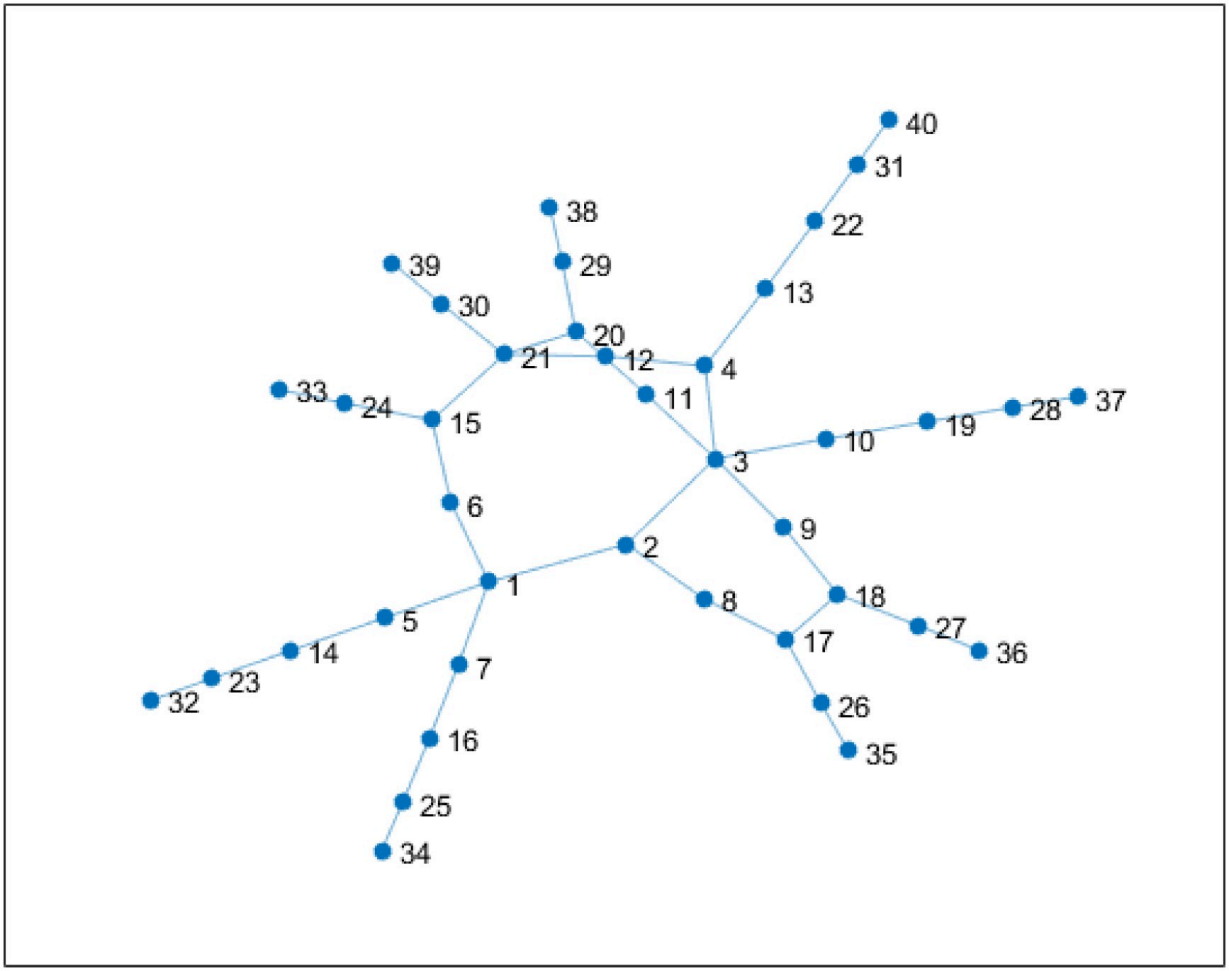
Synthetic network with 40 nodes.

**Fig 5:**
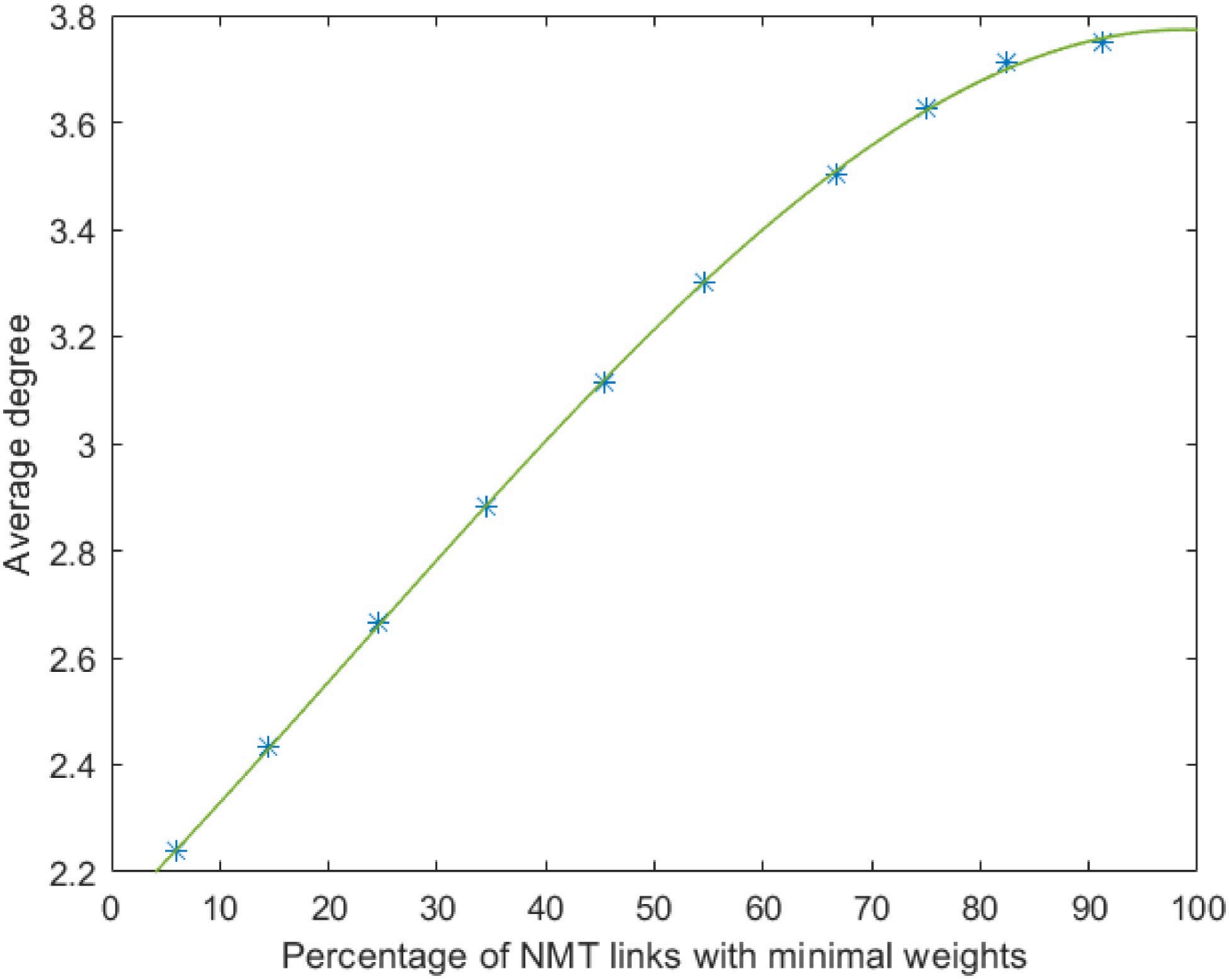
Number of added NMT links.

**Fig 6:**
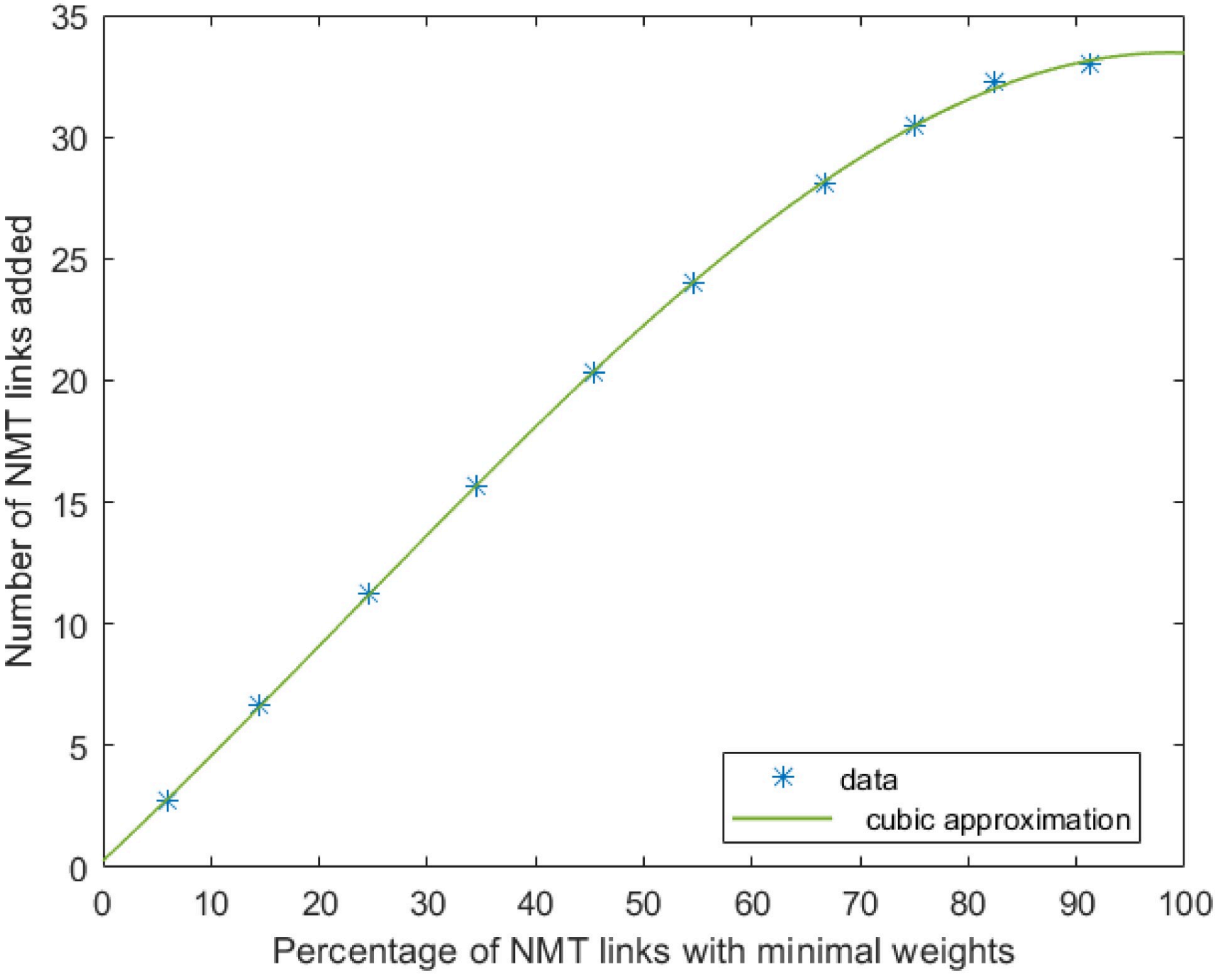
Average degree of the augmented network.

The authors then consider augmentation of the following 40 nodes network with average degree 2.1. (Fig 4). The weights of all edges are set equal to 10. The authors assume that 46 NMT edges are candidate for the network augmentation. *t*_*i*,*j*,*m*_ and *p*_*i*,*j*,*m*_ are randomly selected using a uniform distribution between 1 and 20. *a*_*i*,*j*,*m*_ and *s*_*i*,*j*,*m*_ are also randomly selected but with the constraints $a_{ijm}s_{ijm}\leq \frac{\bar{w}}{t_{ijm}p_{ijm}}.$, $\bar{w}=10$ for a given percentage of links. The links whose weights satisfy this constraints are hereafter called minimal weight NMT links. The authors then evaluate how the number of added edges and the average degree evolve according to the number of minimal weights NMT links. The results depicted in Figs 5 and 6 have been obtained through a Monte-Carlo simulation with 8000 runs. As expected, the number of added NMT links, and consequently the average degree, increases with the number of minimal weight NMT links. In both cases data can be well approximated with third-order polynomials. Therefore by targeting a given average degree, one can set the number of NMT with minimal weight in the set of NMT candidates and then design these edges so that their weights meet the constraint $t_{i,j,m}a_{i,j,m},s_{i,j,m}p_{i,j,m}\leq \bar{w}$.

### Case study: Integrating NMT with bus-train network in Johannesburg, South Africa

While the South African public transportation authorities concur with the global trend associated with green mobility, they nonetheless suffer from a lack of knowledge on how to formalize these green mobility modes, due in part to the limited diversity of methods in existing literature. Historically the public transportation system in the city of Johannesburg, South Africa has been built on scheduled buses, trains and mini-bus taxis. In 2009 and 2010, important expansions were made in the form of the bus rapid transit system (Rea Vaya) [44] and the high speed rail train (Gautrain) [45]. However with regards to NMT (cycling modes), only infrastructure has been put in place, with no formal public transportation system [46].

Given how the city of Johannesburg has identified the Gautrain as the backbone of mobility [44, 45], the Gautrain network can be used to test the possibility of using multi-modes of mobility for commuting trips. It is worth noting that Sandton and Melville (two of Johannesburg’s suburbs) already have tuk-tuks operating without being integrated to the existing formal public transportation services [15]. Moreover, within the South African National Land Transport Act Section 70(1) rickshaws are described as being part of *motor*-*tricycles* classification which may be used for public transport services where relevant transport plans allow for this [15].

#### Study area

The City of Johannesburg, presents a number of unique opportunities when compared to other African cities, with the presence of the rapid railway transit system, being Gautrain, as noticeable difference. Since the launch of this train in 2010, it has taken up a larger mode share of trips within the Gauteng province. Also given how the city has invested in infrastructure for bus rapid transit, the integration of these modes of mobility can greatly reduce traffic congestion and improve travel times for commuters.

The Gautrain being a rapid railway transportation system and the Gaubus being an extension of the Gautrain service in the form of a bus rapid transportation system operates across the City of Johannesburg, City of Tshwane and City of Ekurhuleni. The study focused on routes located within the City of Johannesburg, as it is the economic hub of the Gauteng Province with a population of 4.4 million people and a surface area of 1,645*km*^2^. Currently the Gautrain has 5 operating train stations in Johannesburg:

- Park: it serves as a mobility hub connecting the city with other parts of the country and Africa;
- Rosebank: it hosts South Africa’s biggest company headquarters;
- Sandton: it is currently the fastest growing central business district;
- Malboro: it is spatially located at the heart of one of Johannesburg growing townships Alexandra;
- Midrand: it hosts the newly developed Mall of Africa which attracts shoppers from all over the African continent.

**Fig 7:**
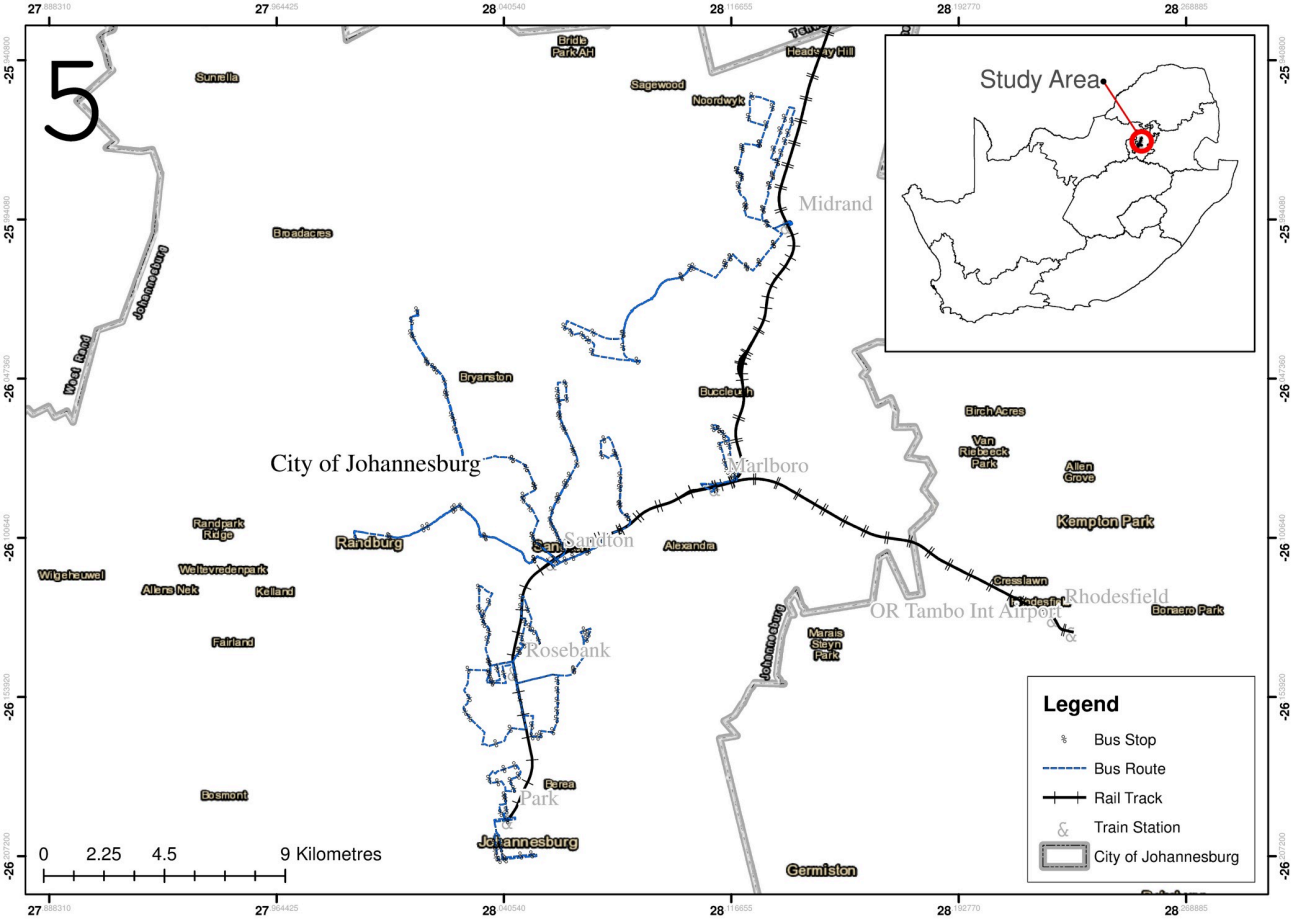
Study area.

Given how the train stations are located at areas of economic importance in the city, the Gaubus was developed with currently 15 routes in Johannesburg as an extension of the Gautrain to transport commuters to and from these points of interest (see Fig 7). Currently commuters in Johannesburg have the option to board the Gaubus at their origin points which will take them to the train station to board the Gautrain. The Gaubus route network from Park to Midrand (see Fig 7) can be represented as a tree graph, with the train stations being hubs.

As it can be seen, the Gaubus network has exactly the structure defined in Fig 1. When a commuter wishes to traverse from any bus stop to the Gautrain station, currently he/she only has one option and if the bus network has a failure/shut down at any point in the network the quality and the feasibility of the commuter’s journey will be severely impacted. Therefore, there is a need for a more robust network providing several alternative routes. Creating new motorized routes could have significant economical and ecological costs. The method described in the previous section is applied to create non-motorized routes to enhance multi-modality of the Gautrain network. Typically, to the existing bus routes and stations, the study aimed at adding green mobility services, non-motorized rickshaw services precisely.

## Results

In this section, description of how to apply the proposed method to the case study is first detailed. Then, evaluation of the properties of the obtained augmented graph with respect to those of the existing one in terms of centrality measures and travel time are provided. Matlab was used to compute the centrality measures.

### Creation of the augmented network

#### Step 1: Build the set *E*_*NMT*_ of candidate edges (short-cuts) by discarding edges with infinite weights

Recall that weights defined in (1) are given by *w*_*ijm*_ = *t*_*ijm*_ *a*_*ijm*_ *s*_*ijm*_ *p*_*ijm*_. For the NMT mode, *a*_*ijm*_ = 1 if the road is adapted to allow circulation of non-motorised rickshaws and if the travel distance is below *D* = 6 km; otherwise *a*_*ijm*_ = ∞. Indeed, rickshaw operators would only be willing to cycle short distances. Moreover, given the limited speed of green mobility modes they turn to only have a competitive advantage to other modes for short trips. Note that the steepness factor is not included here since the considered area is flat.

Due to societal constraints such as safety and crime within the vicinity of Malboro, all possible new edges around Malboro station are penalized by setting *s*_*ijm*_ = ∞ to these edges. For the other locations, *s*_*ijm*_ = 1. Finally, *p*_*ijm*_ = ∞ if there is already an edge between these vertices from the motorized graph; that is if there is already a path (such as the Gaubus route) between them. In situations whereby there are disruptions along the motorized route, set *p*_*ijm*_ = 1. Otherwise, *p*_*ijm*_ = ∞.

**Fig 8:**
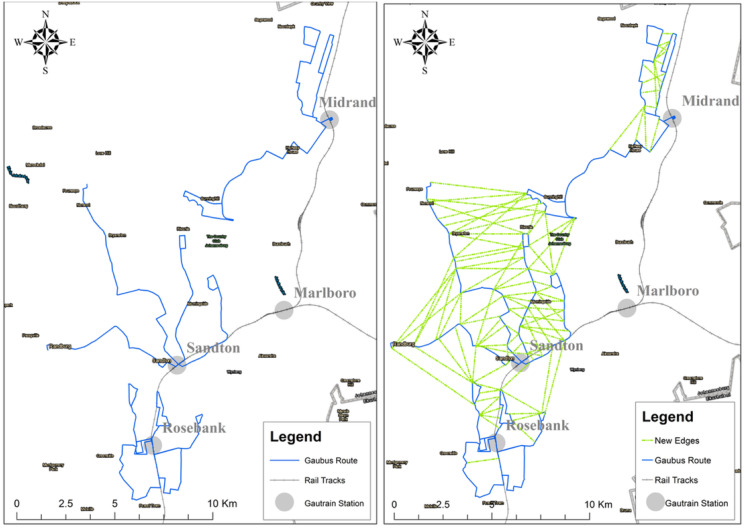
Potential augmented Gaubus network after adding edges in *E*_*NMT*_.

Applying these rules, the authors obtained a set *E*_*NMT*_ with 105 edges as shown in Fig 8

#### Step 2: Computation of the finite weights in *W* and *W*_*NMT*_

**Fig 9:**
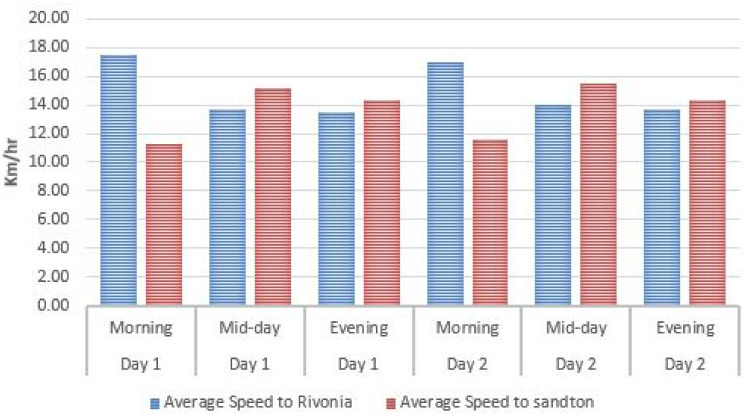
Average travel speed on the S3 Data Gaubus route near Sandton Gautrain station.

**Fig 10:**
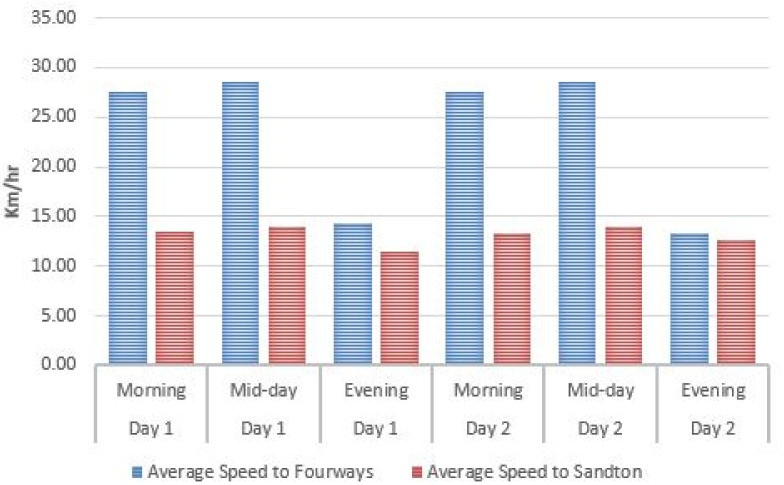
Average travel speed on the S5 Data Gaubus route near Sandton Gautrain station.

**Fig 11:**
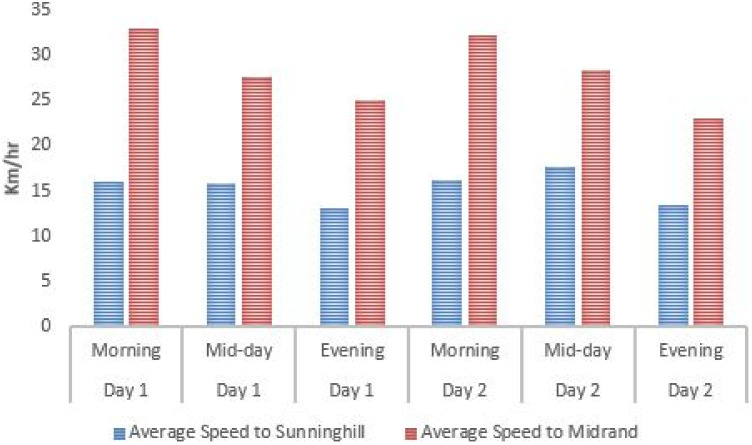
Average travel speed on the M3 Gaubus route near Midrand Gautrain station.

From the first step, one can note that a binary weighting policy was adopted for accessibility, security, and preference parameters. They are either equal to 1 or to ∞. As a consequence *w*_*ijm*_ = ∞ or *w*_*ijm*_ = *t*_*ijm*_; meaning that the only parameters to be computed for finite value weights are *t*_*ijm*_, the travel time. For this purpose, a sample of three bus routes was used, namely the S3 and S5 Datas (both connected to the Sandton Gautrain station) and the M3 (which is connected to the Midrand Gautrain station) to determine the average travel time *T*_*average*_ when using the Gaubus based on the average travelling speed *S*_*average*_ from the first bus stop to the furthest stop as shown on Figs 9–11. Relevant data (spatial location and time) was recorded using a GPS device for two week days for the respective routes during the peak hours (morning from 6am to 8am; mid-day from 12pm to 2pm; and lastly evening from 4pm to 6pm).

From this data set, an average speed of 17.43*Km*/*h* with a standard deviation of 6.56*Km*/*h* is obtained. A result slightly higher than the one in New York [47].

**Fig 12:**
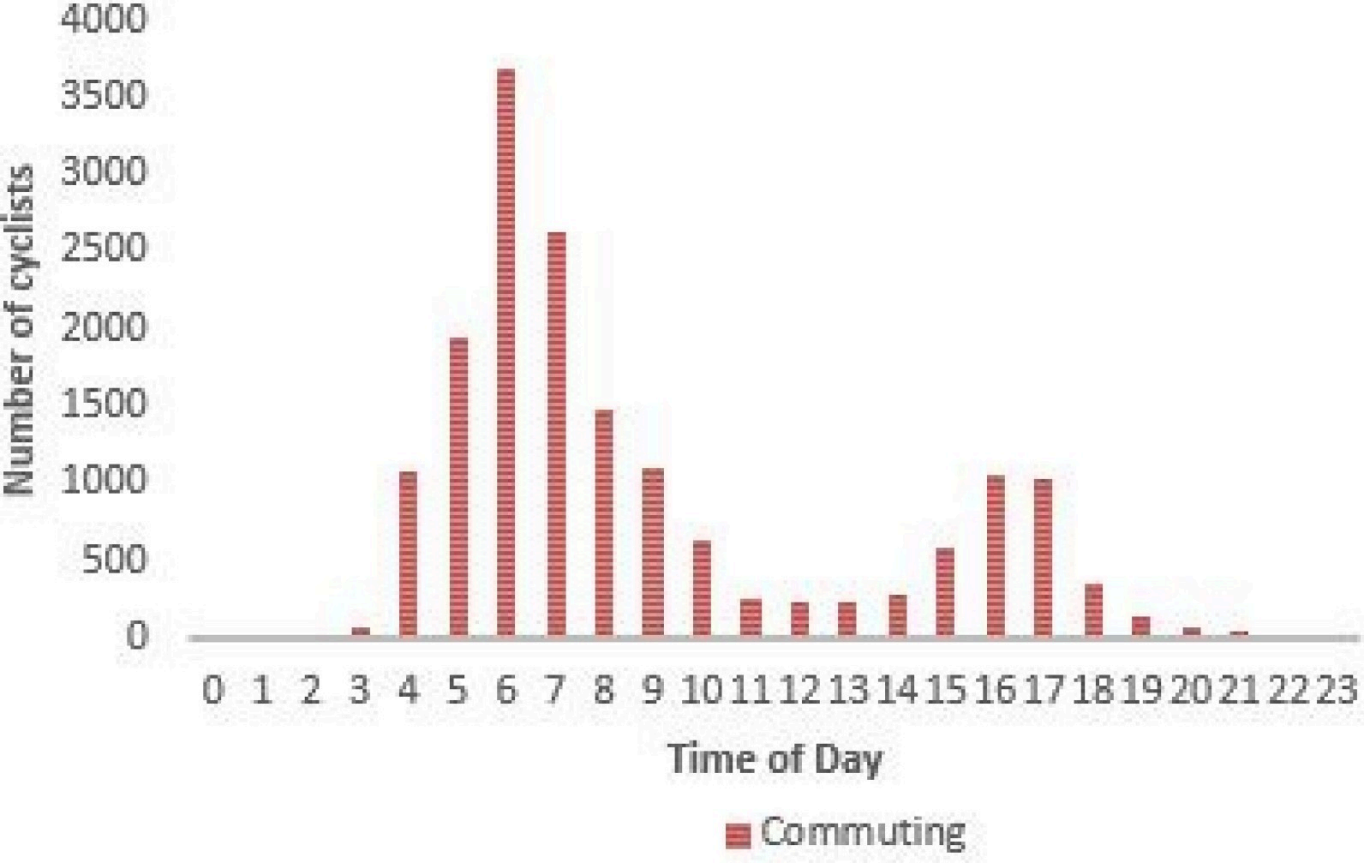
Visualisation of cycling trends based on time of the day.

For NMT mode, cycling data from Strava Metro was used, which provides ground truth on when and where people cycle and run [46]. Using cycling data for the year 2014, the paper computed the *T*_*average*_ of the 16844 cycling commuting trips made within the Johannesburg city region. Fig 12 shows the distribution of cycling commuting trips throughout the day. Most of the cycling commuting trips occurring in early morning and evening recorded an average speed of 15km/h, consequently in the study 15km/h was used to compute *t*_*ijm*_ for the NMT mode.

#### Steps 3 and 4: Computation of a minimum weight spanning tree on the graph *G*∪*G*_*NMT*_, with corresponding weights *W*∪*W*_*NMT*_ and derivation of the augmented network

**Fig 13:**
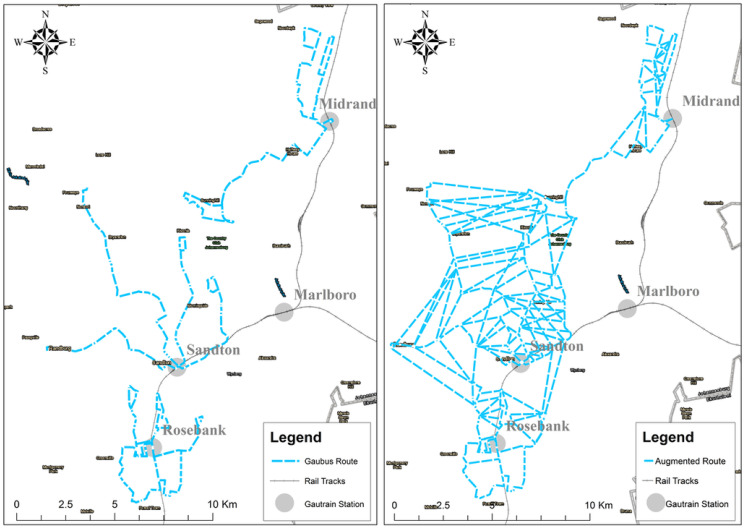
Original network (left) compared to the augmented network obtained with the proposed method (right).

MATLAB was used to compute the minimum cost spanning tree of the graph built in the previous step. Then, the NMT edges of this spanning tree were added to the motorized mode network to give rise to the augmented network depicted in Fig 13.

It is worth mentioning this approach works for simple networks such as for the study area were bus stations only intersects at the location of the train station but also for more complex networks. The challenge would be on the calibration of the weights associated with the considered costs which were supposed to be binary for the case study.

## Discussion

### Analysis of the obtained multi-modal network in terms of centrality metrics

#### Degree centrality

**Fig 14:**
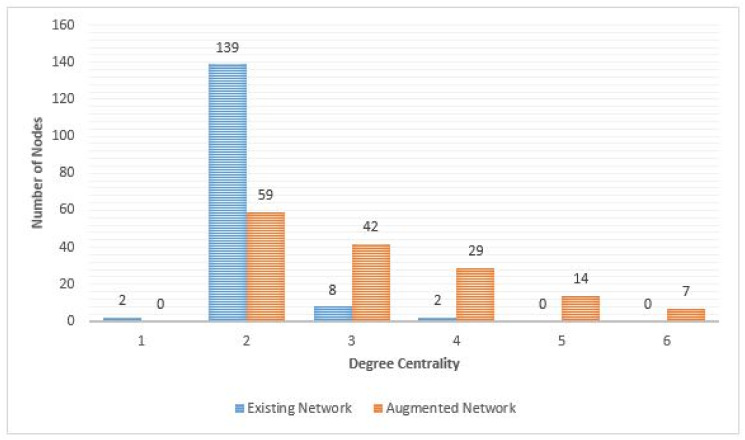
Degree centrality of existing network and augmented network.

The authors first used degree centrality to visualize the importance of each node based on the number of its neighbours. Nodes with many neighbours are considered the most influential. However, having a few high order nodes means if there is a system failure at one of these nodes, commuters will not be able to complete their trip. Hence having an even balance of high order nodes is more desirable. Fig 14 presents the degree centrality of the two networks. One notable aspect is that the augmented graph has evenly distributed degree centrality. The number of nodes having only two neighbours is reduced to 57.55%, meaning that more possibilities for alternative routes are created.

#### Closeness centrality

**Fig 15:**
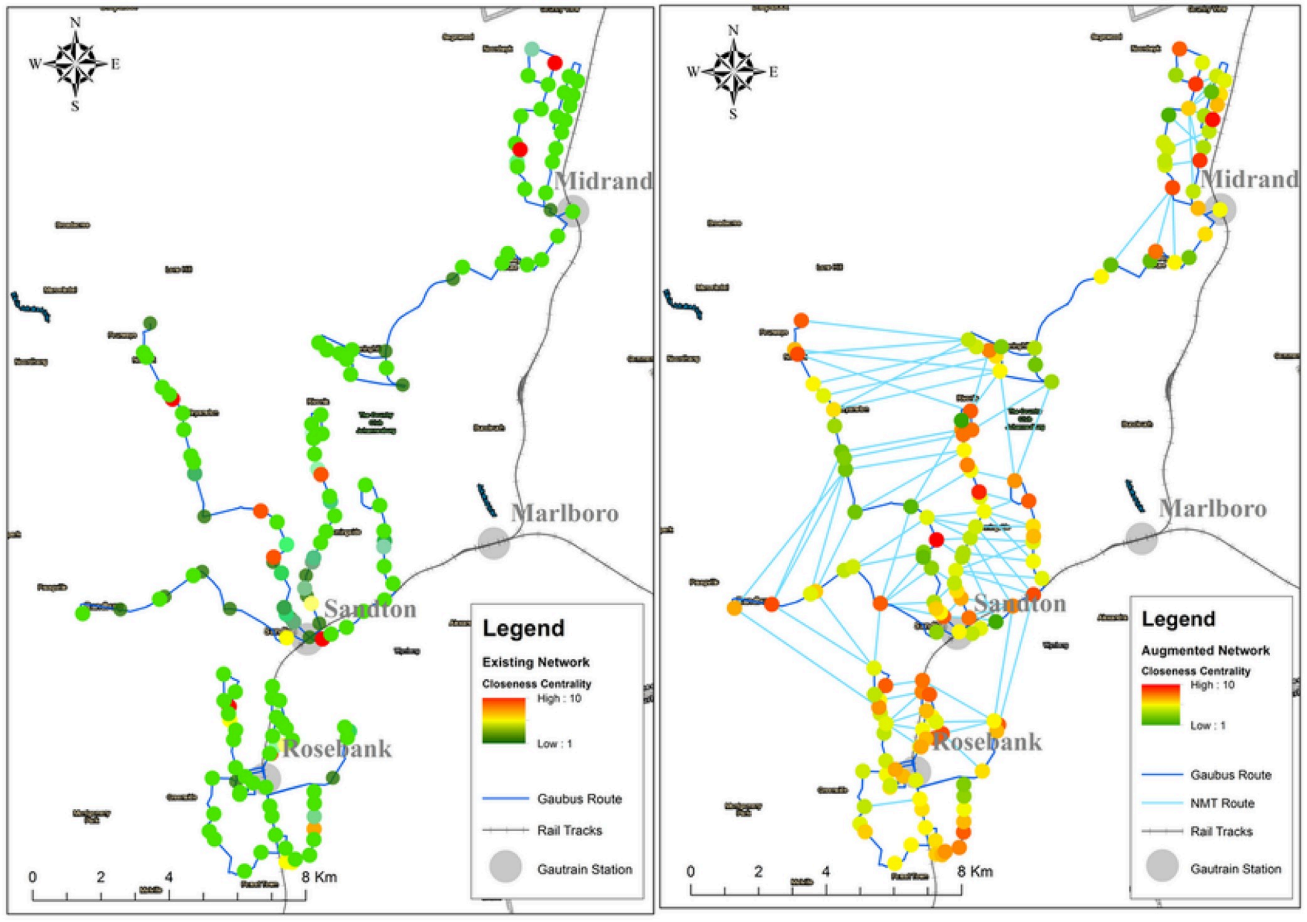
Closeness centrality: Existing network (left) and augmented network (right).

**Fig 16:**
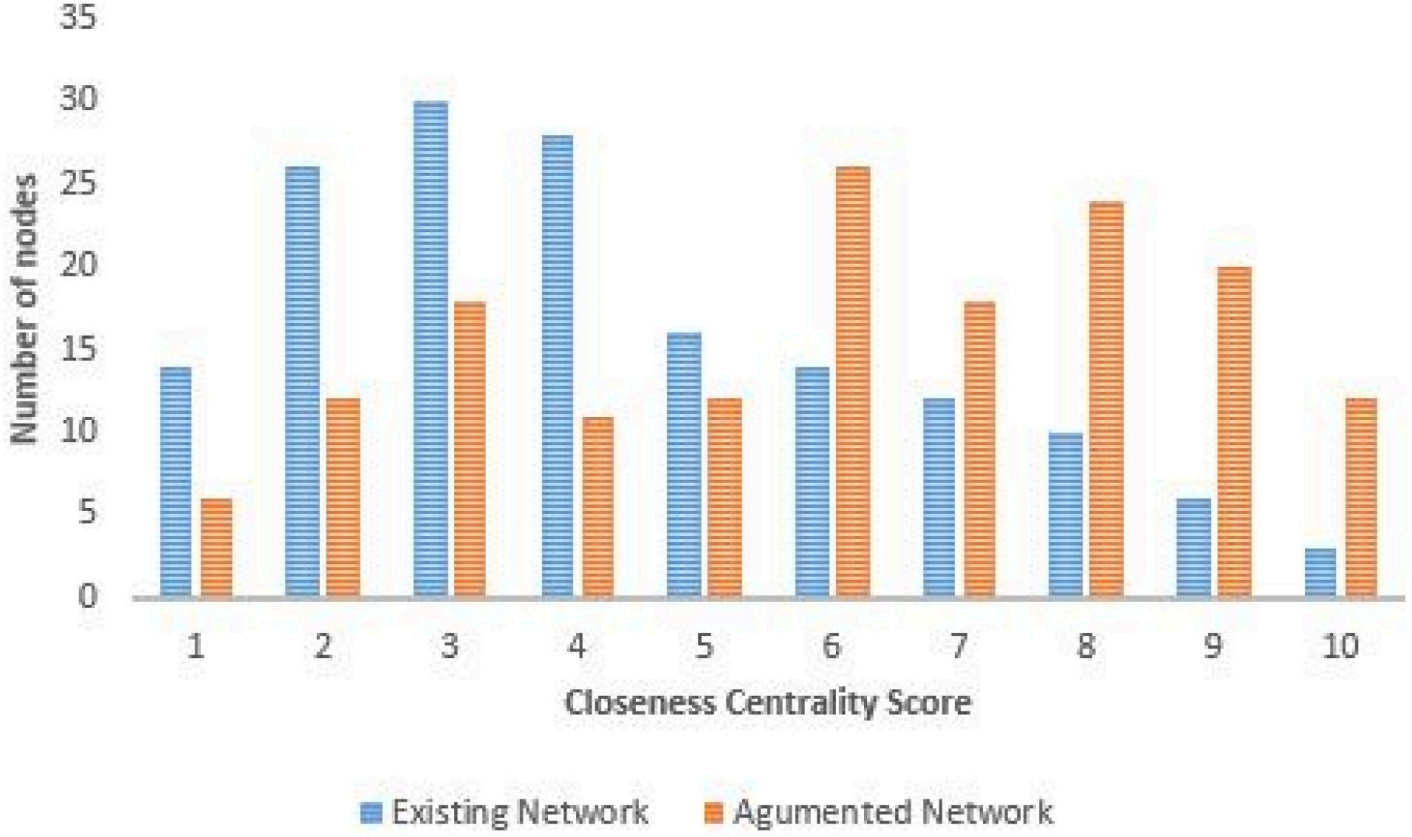
Closeness centrality score: Existing network and augmented network.

Generally nodes with high closeness centrality score are within the path with the shortest distance to all other nodes in the network. Using the reclassification tool in Arc Gis to normalise the results, similarities and differences between the two graphs can be seen in Figs 15 and 16). Using the Jenks natural breaks algorithm in the reclassification tool, class breaks were created between a range of 1 to 10, this allowed for grouping similar values together whilst also maximizing the differences between class ranges Nodes with a score 9 or 10 in this work are refereed to as ‘central nodes’. For the existing network, central nodes are only located near the Gautrain stations, while for the augmented graph there are more additional central nodes, located along the S4 and S5 Datas, M1, M2 and RB3 routes. From comparison of the two graphs the augmented graph has relatively more higher order influential nodes than the existing network graph. The Gaubus routes with the most changes in the nodes were the S2 and S3 Datas located near Sandton Gautrain station; RB3 and RB6 located near Rosebank Gautrain station; M3 and M1 located near Midrand Gautrain station. Based on these results, the introduction of new edges on the graph has led to nodes which previously had a low closeness centrality to become more influential nodes within the system, hence improving the network robustness.

#### Betweeness centrality

**Fig 17:**
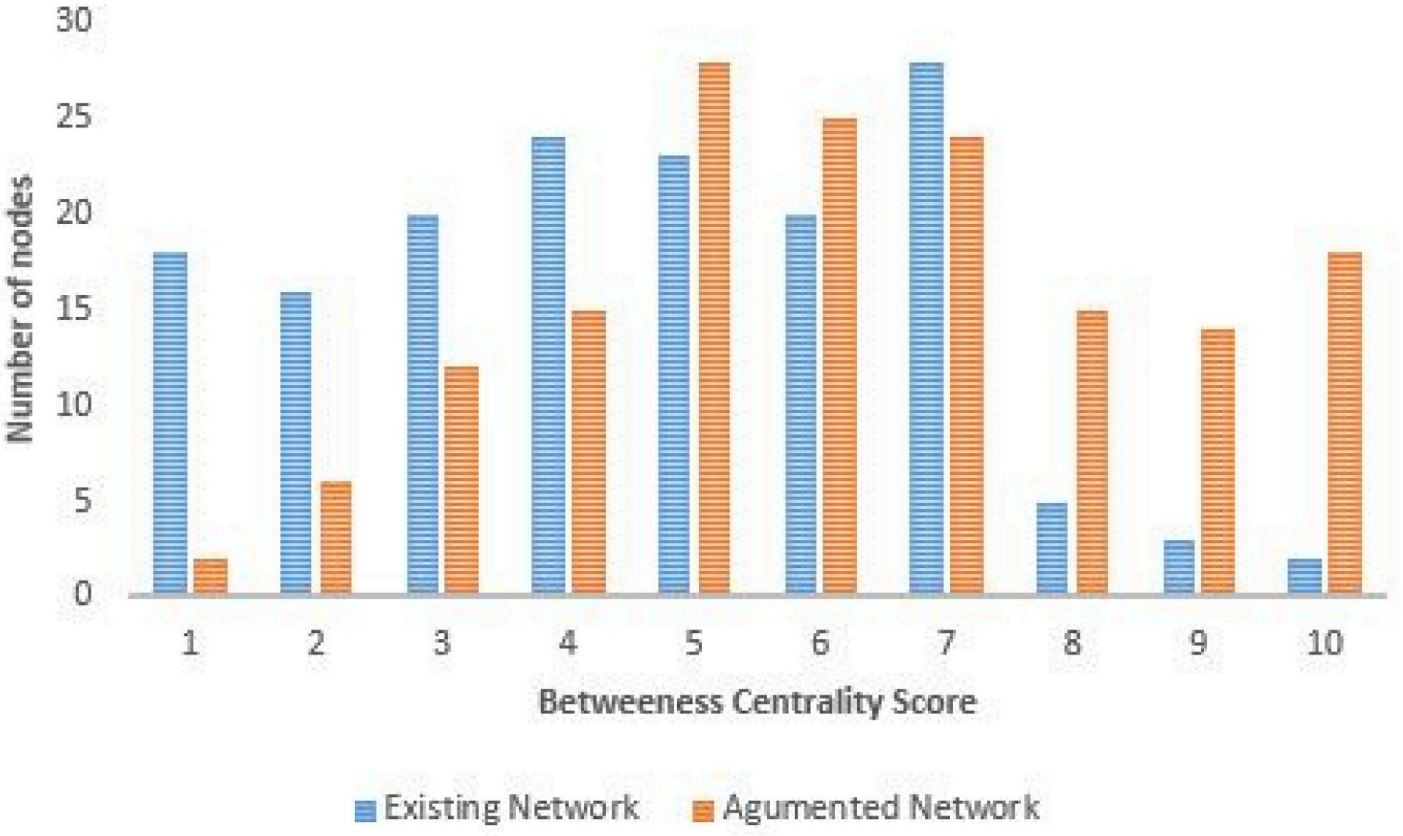
Betweenness centrality score: Existing network vs augmented network.

**Fig 18:**
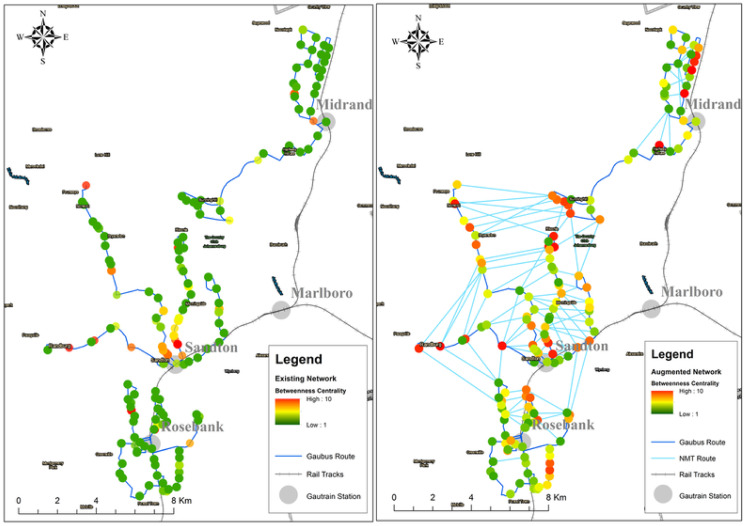
Betweenness centrality: Existing network (left) and augmented network (right).

Likewise for the betweenness centrality analysis, the most influential nodes are different for the two graphs (see Fig 17). The existing network has only a few high order influential nodes and most of these are located near the Gautrain stations. After introducing new edges, the graph had more influential nodes along the various Gaubus routes as shown in Fig 18. One notable change after the introduction of new edges was on the nodes located in Rosebank, these previously had many low order nodes. The introduction of the new edges has increased the nodes spreading ability and consequently promises to reduce commuter travelling time.

### Analysis of the obtained multi-modal network in terms of travel time

Two scenarios are considered as illustration of the gain in terms of travel time improvement.

Scenario 1: A commuter located at Montecasino in Fourways suburb (located along S4 Data route) wants to reach the Sandton Gautrain station during peak hours. With the existing network, the commuter has only one possible route which consists in taking the S4 Data Gaubus. His path would only be possible along the S4 Data Gaubus route. The travel time is approximately 45 to 50 minutes due to traffic congestion on along this path. However, with the creation of new routes, one option could be to use both the Gaubus and a non-motorised rickshaw during the commuting journey. The trip would initiate at the node in Montecasino towards the furthest bus stop along the S3 Data route located in Sunninghill, surburb. Using a non-motorised rickshaw or or green tuk-tuk this would take 14 minutes 6 seconds. The commuter can then take the Gaubus towards Sandton Gautrain station which will take 16 minutes on average. Therefore, the total trip would take a total travel time of 30 minutes 6 seconds; meaning a travel time reduction of 33% to 40%.

Scenario 2: A commuter located at Megawatt Park suburb, in Midrand, wishes to travel to Sandton City. With the existing network, his route would initially be along the M3 Gaubus towards Midrand Gautrain station and then use the Gautrain to travel to Sandton City. The trip duration would be 24 minutes during the peak hour. At peak hours, trains are in general full and the probability to board the train is low and the trip’s comfort could be reduced due to high occupancy. With the introduction of new routes, the commuter can use the new short cuts from the node along the M3 and travel towards the S3 Data Gaubus route. The trip will take 6 minutes 28 seconds using a non-motorised rickshaw or green tuk-tuk. Then, the commuter can board in the Gaubus going to Sandton City which will take on average 16 minutes during peak hours. The total travel time will be 22 minutes 28 seconds. Even though the gain in travel time is only of 8%, the commuter has a high probability to board the train and to make a comfortable trip since current bus occupancy is lower than for the train at peak hours.

### Implications for transportation planning

Contemporary public transportation planning in developing cities such as South Africa seeks to become a sustainable means to ensure economic and social development, although many obstacles still need to be tackled. Currently, knowledge gaps still exist in transportation planning to meet the goal of the Green Transportation Strategy for South Africa (2018-2050) and manage non-motorized infrastructure planning [46]. A notable example is how the City of Johannesburg’s cycle lanes are being used for street parking. Hence, using evidence based research perhaps this work can guide policy on how to integrate this infrastructure with main stream public transportation services in the city.

There is also a mounting pressure to provide affordable public transportation in the face of persistently high fuel costs. As the public is already familiar with using green mobility modes such as tuk-tuks, a campaign to integrate these with main stream public transportation system would be well received. The key in future public transportation in urban cities is the integration of green mobility systems and traditional main stream public transportation modes (buses and trains). However it should be noted that although the bicycle sharing services; green tuk-tuks and rickshaws cannot replace the bus service, they still pose as a viable solution for commuters to use during the morning, afternoon and evening peak hours to reduce total travel time of the commuting trip. This promises many merits in that it enhances the existing mobility network by making it more robust, promotes a healthy living lifestyle for citizens and also seeks to reduce total travel time for commuting trips.

Intergation of NMT and motorized transportation modes can be facilitated in terms of payment in the case of Gautrain network. Currently Gautrain and Gaubus commuters utilize a card boarding system, whereby commuters tag in and out of the Gaubus/train and have the option to reload the card either online or at Gautrain stations. Given how the Gautrain system already has an integrated ticketing system for the train and bus services, expanding the system to include other modes would be advantageous for the whole public transportation network. This integration has the potential to enhance the overall travelling experience as the payment system can be linked the Gautrain mobile app which gives commuters access to real-time travel information regarding timetables and delays on the system. Such a universal payment platforms ensures security and convenience for both the public transportation provider and commuter. Although traditionally rickshaw fares are determined though bargaining, utilization of a predefined rate per distance will reduce on bargaining time, as the commuter would not have to negotiate with several operators before he/she finds one willing to serve them at the desired rate [27]. Additional recharge stations should be placed at the green mobility stands as currently there are no recharge stations at the Gaubus stops. Although commuters can recharge their cards online, providing an additional platform to recharge at bus stops will be advantageous as commuters do not always have access to the internet and this will ensure smooth transfer from one mode to another whilst limiting the need for cash payments.

## Conclusion

This paper has proposed the introduction of new short-cuts or edges to an existing bus route as a means to integrate green mobility modes and traditional public transportation modes. The proposed method consists in solving a graph augmentation problem with respect to a composite cost based on social, geographical and operational constraints. In the case study considered in this paper, the cost associated with these constraints were chosen as binary in order to penalize locations with high level of crime, lack of infrastructure for NMT, or already provided with motorized transportation. In other contexts, different choice of parameters can be more appropriate to include more flexibility. The results, in the South African context, show a significant improvement in term of robustness, integration, and travel-time. The design of the weights of candidate NMT links is an open question. The usage of the proposed composite weight allows four degree of freedom to select and improve weights of NMT links with respect to those of the original network. Indeed, it has been shown in this paper, that the average degree increases when a significant number of NMT links has minimal weights. The optimal weight design problem is definitely to be investigated. Another future work concerns integration of more informal transportation modes, including mini-buses that can be encountered in most developing countries. They are mostly viewed as part of congestion problem while they could be optimized to become part of solution to the mobility problem. Finally, it worth noting that the composite cost defined in this paper could be used for routing problems in order to generate optimal societal-friendly routes adapted to preferences of the user, crime level, noise level, beauty of landscape and other parameters. The cost can also integrate past experiences of commuters through rating as it is the case in nowadays services (restaurants, cabs, hotels, and movies, to cite a few).

## Supporting information

S1 Text (TXT)

S1 Data Bus route data. (XLS)

S2 Data Bus station data. (XLS)

S3 Data Railway track data. (XLS)

S4 Data Train station data. (XLS)

S5 Data route average travel speed data. (XLSX)

S6 Data (XLSX)

S7 Data M3 route average travel speed data. (XLSX)
